# Supplementary material for: Technology-Based Motivation Support for Seniors’ Physical Activity—A Qualitative Study on Seniors’ and Health Care Professionals’ Views
Source: Int J Environ Res Public Health. 2019 Jul 8;16(13):2418. doi: 10.3390/ijerph16132418 (PMC6651538; doi:10.3390/ijerph16132418)
Supplement: Supplementary File 1 [file ijerph-16-02418-s001.zip › IJERPH Appendix E 20190705.docx]

**Appendix E *–* Similarities and differences between seniors’ and HCPs’ views on how digital technology could contribute to supporting and motivating seniors to increase PA**

| Contributions identified through analysis of seniors’ and HCPs’ views | | Seniors’ and HCP’s’ views on contributions* | |
| --- | --- | --- | --- |
| *Identified contribution* | *Sub-contribution* | *Sub-category senior* | *Sub-category HPC* |
| Making PA more enjoyable | Strengthening social interaction | Supporting social interaction | Stimulating social interaction |
|  | PA through game and fun | Containing playful elements | Making PA more fun |
| Providing support | Supporting coaching of PA | Providing personal coaching | Supporting communication |
|  |  |  | Supporting follow-up at a distance |
|  |  |  | Providing reminders |
|  | Fitting organizations that could provide coaching | Used within associations | Supporting the clinical work |
|  |  | Prescribed by HCPs | Fulfilling requirements from health care organization |
|  | Coming with support | Support available | Further support and customization for patients with specific needs |
| Strengthening the seniors’ control | Educating in PA |  | Pushing habit change |
|  |  |  | Increasing knowledge/insight on PA |
|  |  |  | Providing visual guidance |
|  |  |  | Preventing passivity |
|  | Supporting self-monitoring | Informing on current activity level | Providing objective activity data |
|  |  |  | Supporting checking off performed PA |
|  |  |  | Supporting setting of functional goals |
|  |  |  | Supporting setting individual and flexible goals |
|  |  |  | Clarifying risk behavior |
|  |  | Acknowledging progress | Rewarding decreased inactivity |
|  |  |  | Strengthening the senior's engagement |
| Contribute to well-being | Feeling modern |  | Attractive design (making users feel modern) |
|  | Confidence |  | Communicating results in a suitable way |
|  |  |  | Credible and easy to recommend |
|  | Supportive | Supportive | Making seniors feel better and see progress |
|  |  | Non-demanding | Easy to use and understand |
|  | Safety | Does not provoke fear |  |
|  |  | Confirming physiological signals | Decrease fear and doubt |
|  | Enjoyment |  | Make both PA and technology use fun |

* Sub-categories from inductive analysis of focus group interviews with seniors and HCPs, respectively
