# Supplementary material for: Technology-Based Motivation Support for Seniors’ Physical Activity—A Qualitative Study on Seniors’ and Health Care Professionals’ Views
Source: Int J Environ Res Public Health. 2019 Jul 8;16(13):2418. doi: 10.3390/ijerph16132418 (PMC6651538; doi:10.3390/ijerph16132418)
Supplement: Supplementary File 1 [file ijerph-16-02418-s001.zip › IJERPH Appendix F 20190705.docx]

Appendix F – Similarities and differences between seniors’ and HCPs views on qualities of the technology-based digital support

| Qualities identified through analysis of seniors’ and health care professionals’ views | Seniors’ and health care professionals’ views on qualities the technology should have*  *Sub-category Senior* *Sub-category HCPs* | |
| --- | --- | --- |
| *Identified qualities* |  |  |
| User-friendly | Smooth and simple | Easily carried around |
|  |  | Safe and smooth data management |
| Reliable | Robust | Functioning stably |
| Customizable | Modular | Individualizable (disabilities, languages, aim of PA, needs, preferences, situation) |
| Interoperable | Possible to integrate | Interoperable and usable without WiFi home |
| Effective for the purpose | Facilitating PA in daily life | Having a clear aim and long-term effect on PA |
| Accessible in a motivating way |  | Motivating accessibility with no/small cost |

* Sub-categories from inductive analysis of focus group interviews with seniors and HCPs, respectively
