# Supplementary material for: Technology-Based Motivation Support for Seniors’ Physical Activity—A Qualitative Study on Seniors’ and Health Care Professionals’ Views
Source: Int J Environ Res Public Health. 2019 Jul 8;16(13):2418. doi: 10.3390/ijerph16132418 (PMC6651538; doi:10.3390/ijerph16132418)
Supplement: Supplementary File 1 [file ijerph-16-02418-s001.zip › IJERPH appendix C 20190705.docx]

**Appendix C – Seniors’ views on digital technology for supporting and motivating seniors to increase PA**

| **Theme** | **Category** | **Sub-category** |
| --- | --- | --- |
| A help for the user in daily life | Surmountable | Smooth and simple  Robust  Non-demanding  Does not provoke fear  Support available |
|  | Customizable | Possible to integrate  Modular |
|  | Helpful facilitator | Supportive  Facilitating PA in daily life |
| Strengthening motivation for PA, also among inactive persons | Conscious-raising | Informing on current activity level  Acknowledging progress  Confirming physiological signals |
|  | Making physical activity enjoyable | Supporting social interaction  Containing playful elements  Providing personal coaching |
|  | Useful for organizations reaching inactive persons | Prescribed by health care professions  Used within senior associations and health clubs |
