# Supplementary material for: Technology-Based Motivation Support for Seniors’ Physical Activity—A Qualitative Study on Seniors’ and Health Care Professionals’ Views
Source: Int J Environ Res Public Health. 2019 Jul 8;16(13):2418. doi: 10.3390/ijerph16132418 (PMC6651538; doi:10.3390/ijerph16132418)
Supplement: Supplementary File 1 [file ijerph-16-02418-s001.zip › IJERPH Appendix B submitted.docx]

Appendix B – Interview guide for focus group with health care professionals

*Introduction of the participants to the focus group discussion*

Welcome the participants to the University and thank them for participating.

The discussion leader and assessor introduce themselves and the project, go through the information to the participants and the form for written consent. Explain the aim and procedure for the audio recording was explained as well as how the results will be used. Explain confidentiality and result reporting on group level so that no results can be traced to an individual. Explain the procedure of the discussion: first a round and then participants are free to express their views under moderation of the discussion leader. Explain the role of the assessor (notes and conclusions)

*Introduction of the participants to the discussion subject*

The main purpose of the technology is to stimulate seniors to increase their physical activity. It should be possible for the seniors to manage independently in their home. The technology should provide the user with information on how physically active he/she has been. We envision that the physical activity measured should relate to a goal that the senior user has formulated. This goal should be able to customize with regards to the individual’s current capacity and well-being. Setting the goal could take into account the person’s recently performed daily physical effort. The technology should comprise a small monitor of daily physical activity, for example embodied in a clock, bracelet or brooch. The monitor could in itself provide the user with information on amount of activity measured. Users that are interested in receiving more comprehensive information about the measurements could access that in a software used on a personal computer, smartphone or a novel type of interaction device.

*Opening questions*

Could you please say something about yourself and how your professional role relates supporting and motivating seniors to increase their physical activity? How do you see technology as an aid in your work to support and motivate older persons to increase their physical activity?

Do you see that technology that monitors physical activity can contribute any value for your work with older persons?

*Transition question*

In what way do you see that you can support older persons to increase their physical activity (and decrease their inactivity)?

What do see as the main challenge in the work on motivating seniors to increase their physical activity?

Can you see any possibilities/benefits for technology supporting seniors’ physical activity? (For example, technology monitoring the seniors’ physical activity, inactivity and execution of different motions)

What would motivate you to use technology in your work?

Do you see hinders/challenges in your use of technology in your work?

*Key questions*

What is your view on the potential of technology to fulfil needs that could facilitate or improve your work to stimulate seniors to increase their physical activity or to carry out training activities that you have recommended?

How could results provided by the technology contribute to your work?

What would make the technology attractive to you?

What needs do you see for increasing older adults’ physical activity?

What could contribute to making the technology useful for you in your work?

What could contribute to making the technology practically usable for you?

In order to be useful for your working role: What would must the technology do? What must it not do?

Are there requirements on the technology’s performance and quality? I. e. What must the technology be able to manage?

Does the organization you work in have requirements on the technology?

What advice do you have for persons developing technology supporting your work on stimulating and motivating seniors to increase their physical activity?

*Terminative question*
Based on what we have discussed today, what is the most important thing you would like to emphasize related to older adults and motivational technology for physical activity supporting your professional role?

*Introduction of the participants to the focus group discussion*

Thank the participants for attending and contributing to the discussions.
