# Supplementary material for: Technology-Based Motivation Support for Seniors’ Physical Activity—A Qualitative Study on Seniors’ and Health Care Professionals’ Views
Source: Int J Environ Res Public Health. 2019 Jul 8;16(13):2418. doi: 10.3390/ijerph16132418 (PMC6651538; doi:10.3390/ijerph16132418)
Supplement: Supplementary File 1 [file ijerph-16-02418-s001.zip › IJERPH Appendix D 20190705.docx]

**Appendix D – HCPs’ views on digital technology for supporting and motivating seniors to increase PA**

| **Theme** | | **Category** | **Sub-category** |
| --- | --- | --- | --- |
| A tool for strengthening the seniors’ own motivation and ability to perform PA | | Increasing the seniors’ direct motivation for PA | Increasing knowledge/insight on PA  Stimulating social interaction  Making PA more fun  Supporting self-registration of performed PA  Supporting setting of functional goals  Preventing passivity |
|  |  | Helping the seniors overcome hinderances to PA | Providing visual guidance  Providing reminders  Decreasing fear and doubt |
|  |  | Increasing the senior’s indirect motivation for PA through decreased inactivity | Clarifying risk behavior  Pushing habit change  Rewarding decreased inactivity  Communicating results in a suitable way |
| Useful both for health care professionals and seniors | | Attractive for seniors | Making seniors feel better and see progress  Making both PA and technology use fun  Easy to use and understand  Attractive design (making users feel modern)  Easily carried around  Functioning stably  Individualizable (disabilities, languages)  Safe and smooth data management  Interoperable and usable without WiFi home  Motivating accessibility with no/small cost |
|  |  | Supporting the HCPs clinical work | Having a clear aim and long-term effect on PA  Credible and easy to recommend  Customizable (according to aim of PA, needs preferences, situation)  Supporting setting individual and flexible goals  Fulfilling requirements from the health care organization  Further support and customization for patients with special needs |
|  |  | Facilitating dialogue and co-operation between senior and health care professional | Supporting communication  Strengthening the seniors’ engagement  Providing objective activity data  Supporting follow-up at a distance |
|  |  |  |  |
