# Supplementary material for: Technology-Based Motivation Support for Seniors’ Physical Activity—A Qualitative Study on Seniors’ and Health Care Professionals’ Views
Source: Int J Environ Res Public Health. 2019 Jul 8;16(13):2418. doi: 10.3390/ijerph16132418 (PMC6651538; doi:10.3390/ijerph16132418)
Supplement: Supplementary File 1 [file ijerph-16-02418-s001.zip › IJERPH Appendix A submitted.docx]

Appendix A – Interview guide for focus group with seniors

*Introduction of the participants to the focus group discussion*

Welcome the participants to the University and thank them for participating.

The discussion leader and assessor introduce themselves and the project, go through the information to the participants and the form for written consent. Explain the aim and procedure for the audio recording was explained as well as how the results will be used. Explain confidentiality and result reporting on group level so that no results can be traced to an individual. Explain the procedure of the discussion: first a round and then participants are free to express their views under moderation of the discussion leader. Explain the role of the assessor (notes and conclusions)

*Introduction of the participants to the discussion subject*

The main purpose of the technology is to stimulate seniors to increase their physical activity. It should be possible for the seniors to manage independently in their home. The technology should provide the user with information on how physically active he/she has been. We envision that the physical activity measured should relate to a goal that the senior user has formulated. This goal should be able to customize with regards to the individual’s current capacity and well-being. Setting the goal could take into account the person’s recently performed daily physical effort. The technology should comprise a small monitor of daily physical activity, for example embodied in a clock, bracelet or brooch. The monitor could in itself provide the user with information on amount of activity measured. Users that are interested in receiving more comprehensive information about the measurements could access that in a software used on a personal computer, smartphone or a novel type of interaction device.

*Opening questions*

Could you please say something about yourself and your view on physical activity? (What supports and motivates you to be active? What can be hinders or challenges for your activity?)

What do you think when we say “technology supporting and motivating older persons’ physical activity”?

*Transition question*

In what way can you see that older persons need support for increasing their physical activity (and decrease their inactivity)?

*Key questions*

What’s your view on how technology could contribute to supporting and motivating older adults to increase their physical activity? What requests and hopes do you thing the older users have?

What needs do you see for increasing older adults’ physical activity? What needs do you see for increasing older adults’ motivation for being physically active?

What do you think could contribute to making the technology attractive for older persons (i.e. supporting the usage of the technology and contributing to positive feelings and well-being)? What do you think would be the seniors’ goal for using the technology?

How would you like to be motivated by the technology?

Which qualities do you think are prerequisites of technology for older users? Which requirements do you think that the technology must meet? What must the technology manage?

What qualities must wearable monitors possess in order to suit older users?

How you think handling digital devices containing screens (personal computer, tablet, mobile phones) suits older persons?

What difficulties do you see related to older users’ possibilities in using technology?

Do you have examples of technical devices that you perceive as useful and manageable?

What should the technology do? What should the technology not do?

What advice would you like to give to persons developing technology for older users?

*Terminative question*

Based on what we have discussed today, what is the most important thing you would like to emphasize related to older adults and motivational technology (that could support increased physical activity)?

*Introduction of the participants to the focus group discussion*

Thank the participants for attending and contributing to the discussions.
